# Fingolimod‐associated Balo's concentric sclerosis in multiple sclerosis: A case report

**DOI:** 10.1002/ccr3.9266

**Published:** 2024-08-06

**Authors:** Parisa Sharifi, Amir Moradi, Abdorreza Naser Moghadasi

**Affiliations:** ^1^ Multiple Sclerosis Research Center, Neuroscience Institute Tehran University of Medical Sciences Tehran Iran; ^2^ Atherosclerosis Research Center Ahvaz Jundishapur University of Medical Sciences Ahvaz Iran

**Keywords:** Balo's concentric sclerosis, fingolimod, multiple sclerosis, tumefactive demyelinating lesions

## Abstract

A report of Balo's concentric sclerosis developed alongside with fingolimod use in a patient with previously diagnosed multiple sclerosis.

## 1 INTRODUCTION

Multiple sclerosis (MS) is a chronic autoimmune disease that primarily affects women and young adults and characterized by demyelination of the central nervous system.^1^ Current management approaches are concentrated on symptom relief, acute attack treatment, and lowering biological activity through disease‐modifying therapies (DMT).^2^ The first oral DMT licensed for the treatment of MS is fingolimod.^3^ Atypical demyelinating disorders exhibiting tumor‐like features present significant diagnostic and therapeutic challenges for neurologists. Tumefactive demyelinating lesions (TDLs) are rare tumor‐like lesions in the central nervous system presenting diverse symptoms that predominantly affect motor, cognitive, sensory, cerebellar, and brainstem functions.^4^ Balo's concentric sclerosis (BCS) emerges as an uncommon variant of TDLs, distinguished radiologically by a “tree trunk” or “onion bulb” appearance on magnetic resonance imaging (MRI), characterized by alternating bands of myelin preservation and loss around them.^5^, ^6^ Despite TDLs being observed in MS patients on fingolimod,^7^ documentation on Balo‐like lesions remains limited. Herein, we present a young female MS patient treated with fingolimod who was admitted to our clinic with hemiparesis and a Balo‐like lesion in her MRI.

## 2 CASE HISTORY/EXAMINATION

A 37‐year‐old female with a 9‐year history of relapsing–remitting MS (RRMS) and 3‐year fingolimod treatment presented to Sina Hospital's emergency department in Tehran, Iran, exhibiting acute right‐sided hemiparesis persisting for 5 days. The patient had no complaints of fever, headache, diplopia, imbalance, or aphasia. She was alert and oriented at admission, and her vital signs were stable. Her pupils were normal in size and had a normal reaction to light. Eye movements were normal in all directions, with no observed ptosis, lid lag, or nystagmus. A physical examination of muscles revealed normal tone in upper and lower limbs on both sides and a medical research council score of 3/5 for proximal and distal muscles of the right upper and lower limbs and 4/5 for proximal and distal muscles of the left upper limb and 5/5 for proximal and distal muscles of the left lower limb. Deep tendon reflexes were assessed using a reflex hammer on the biceps, triceps, brachioradialis, patellar, and Achilles tendon. The reflex responses were normal on the left side and increased on the right side.

## 3 METHODS

**FIGURE 1:**
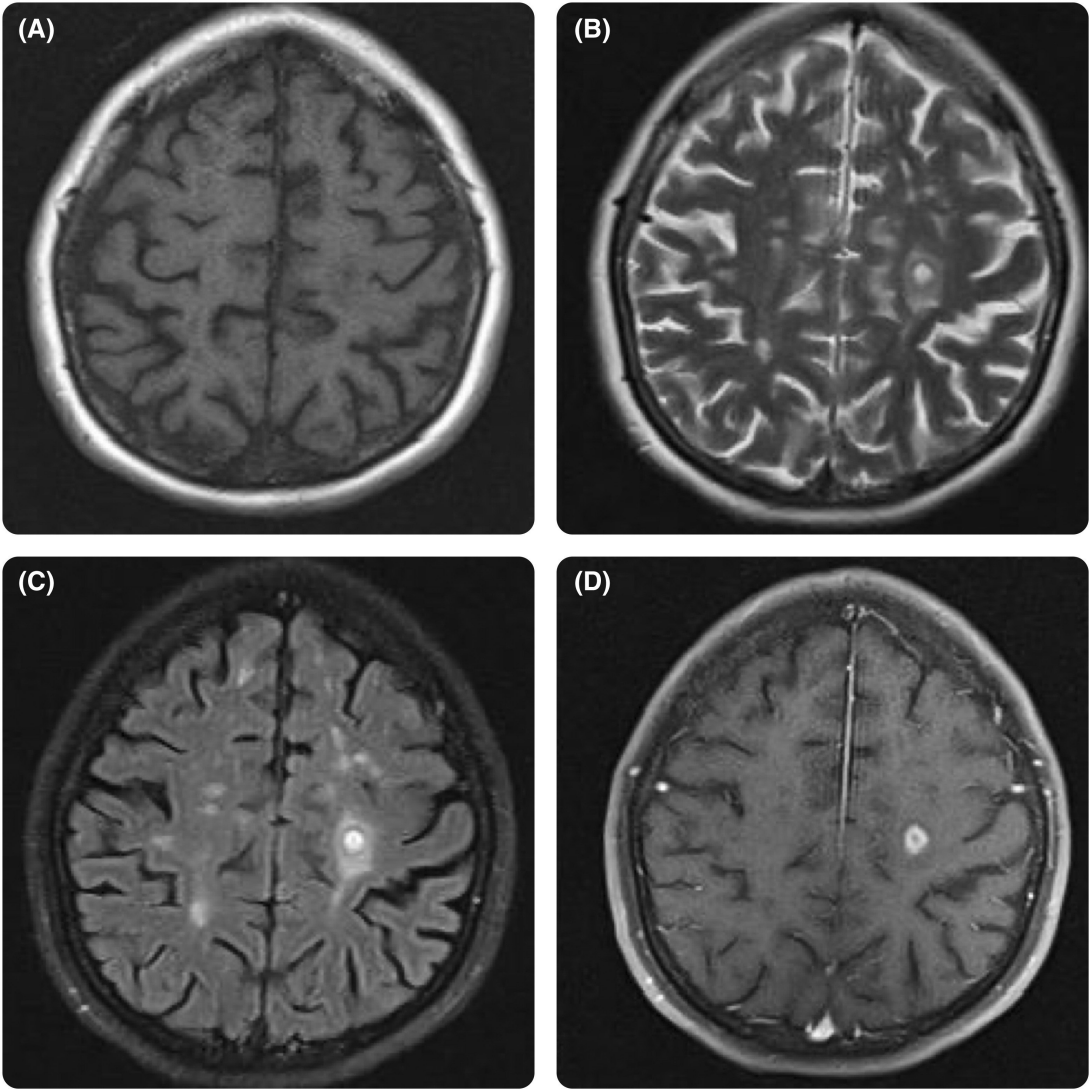
(A) Brain MRI T1‐weighted image showed multiple hypointense lesions. (B, C) T2‐ and flair revealed multiple hyperintense lesions. One of them located in left centrum semiovale had multiple hypo‐ and hyperintense layers, (D) with closed ring enhancement. MRI, magnetic resonance imaging.

A brain MRI was requested, revealing closed ring and target lesions in the left parietal lobe, prompting consideration of infectious etiologies (Figure 1). Subsequent lumbar puncture yielded normal cerebrospinal fluid (CSF) biochemistry results (WBC: 0, RBC: 2, glucose: 69 mg/dL, and protein: 16 mg/dL). Additional studies on CSF, including tuberculosis, brucella, cryptococcus, wright, coombs wright, venereal disease research laboratory, and angiotensin‐converting enzyme, were negative. Moreover, CSF cytologic assessments did not reveal any signs of malignant cells. Patient had anemia and lymphopnia on complete blood count assessment (Hb: 9.2 gr/dL, WBC: 4.4 × 10^3^/μL, lymphocyte: 12.7%, and neutrophil: 84.3%). Further laboratory examinations, including renal and liver function tests, erythrocyte sedimentation rate, C‐reactive protein, blood sugar, arterial blood gas, toxoplasmosis, JC virus, urine analysis, urine culture, stool exam, stool culture, and serum electrolytes, did not yield any pathologic findings. Viral markers, including hepatitis B core antibody, hepatitis B surface antigen, hepatitis C virus antibody, and human immunodeficiency virus antibody, were nonreactive. Additional abdominopelvic CT scan was performed to assess any signs of malignancy or metastases, which did not reveal any pathologic findings.

## 4 CONCLUSION AND RESULTS

With infectious causes ruled out, fingolimod‐induced BCS emerged as the leading diagnosis. As the BCS could be caused by fingolimod, the drug was discontinued, and 1 g/day of IV methylprednisolone was administered for 5 days, resulting in improved limb strength by the fifth day of corticosteroid therapy. Subsequently, rituximab was initiated 15 days after fingolimod cessation. We followed the patient monthly and she exhibited improvement in symptoms, with the absence of any new focal neurological deficits noted during 8‐month follow‐up assessments.

## 5 DISCUSSION

In this case report, we describe a young patient diagnosed with MS who had been under treatment with fingolimod for 3 years. The patient presented to our clinic exhibiting symptoms of hemiparesis, and upon examination, we detected a Balo‐like lesion in her MRI scan without any symptomatic indications of infection.

Fingolimod is an unselective modulator of the sphingosine‐1‐phosphate (S1P) receptor and is commonly used by clinicians to manage relapsing forms of MS (RRMS).^2^ It is generally well‐tolerated, while side effects might include mild abnormalities in standard laboratory evaluations,^8^, ^9^ heart block, bradycardia, macular edema, as well as infections including disseminated varicella‐zoster virus and cryptococcal infections.^10^ Notably, patients with MS receiving fingolimod treatment may develop TDLs even after several years.^7^ TDLs are lesions greater than 2 cm on T2‐weighted brain MRI that can be similar to tumors.^11^ Although TDLs can be observed in various diseases such as autoimmune‐mediated encephalitis, acute disseminated encephalomyelitis (ADEM), myelinoclastic diffuse sclerosis (Schilder's disease), neuromyelitis optica spectrum disorder (NMOSD), and BCS,^5^ MS represents the most prevalent association.^12^

Several hypotheses exist regarding the underlying mechanisms of TDL formation induced by fingolimod in MS patients. It has been suggested that immune cell redistribution occurs in susceptible MS patients, with effector CD8+ T cells, a subset of cytotoxic cells, being particularly implicated. These effector CD8+ T cells can release perforin, thereby directly damaging tissue.^11^ In a case report by Pilz et al., a patient with recurrent TDLs who was treated with fingolimod had a higher concentration of CD8+ T cell in the CSF than in the peripheral blood.^13^

Another theory suggests that individuals with impaired adaptive cell immunity may have an overreaction of the innate immune system as a compensation mechanism. Cytokine effects rather than direct T‐ or B‐cell immunotoxicity may underlie the vasogenic edema and macrophage activation observed in TDLs, thus elucidating the occurrence of these lesions in lymphopenic patients.^11^, ^14^

BCS represents a severe monophasic demyelinating disorder, classified as a rare subtype of MS.^15^ Patients typically present with focal neurological deficits, headache, dizziness, poor cognition, and convulsions.^16^ BCS is characterized by a lesion with demyelination rings mixing with undamaged myelin rings.^17^ Lesions have been reported in the brain stem, spinal cord, cerebellum, hemispheres, and optic chiasm.^18^ Treatment approaches for BCS are still controversial; however, there are several treatment options for BCS. Corticosteroids are primarily used for acute BCS lesions, while Interferon beta‐1a, mitoxantrone, natalizumab, and rituximab are utilized for maintenance treatment.^19^ Rituximab has been considered effective for patients who had BCS with typical MS lesions and responded poorly to steroids, indicating it could be a suitable treatment option in these cases. However, it highlights the need for personalized treatment approaches based on individual patient characteristics and response to initial therapies.^6^ In addition to BCS, some infections of the central nervous system can cause brain lesions with ring enhancement patterns that look like those found in BCS, including herpes simplex virus, cytomegalovirus, hepatitis C virus, tuberculosis, syphilis, cryptococcus neoformans, and pneumocystis jirovecii.^20^, ^21^, ^22^ As mentioned earlier, TDLs constitute a range of demyelinating illnesses, of which BCS is considered a rare subvariant of TDLs.^23^ However, TDLs and BCS can differ in appearance, etiology, and relationship to MS. TDLs are characterized by their large size (usually >2 cm) and atypical features, including edema, open ring enhancement on MRI, and mass effect. On the other hand, BCS is characterized by its specific pattern of demyelination on T2‐weighted MRI sequences, radiologically as two or more concentric rings of alternating hyperintensity and hypointensity.^24^ From an etiological perspective and their relationship to MS, BCS is seen as a distinct disorder or a variety of MS, but TDLs can develop in people with different demyelinating diseases, which does not necessarily include MS.^5^, ^12^, ^15^

The case described herein involves a known MS patient who had been undergoing fingolimod therapy for 3 years preceding the identification of a Balo‐like lesion on MRI. A comprehensive diagnostic evaluation excluded alternative diagnoses mimicking Balo‐like lesions, including intracranial neoplasms and infectious etiologies. There is a growing body of literature suggesting a possible association between fingolimod therapy and the development of new TDLs in MS patients,^7^, ^13^, ^25^ In a study by Baghbanian et al., a 32‐year‐old MS patient receiving fingolimod treatment who acquired Balo‐like lesions was described. She had symptoms such as fatigue, uncontrolled crying, and paralysis of the left side, but testing revealed no indication of cancer or metastases. The patient responded dramatically to corticosteroid pulse therapy.^7^ Nevertheless, reports of Balo‐like lesion development in MS patients receiving fingolimod remain largely anecdotal. Furthermore, while BCS is usually considered to be a precursor to MS, the occurrence of new BCS lesions in MS patients undergoing fingolimod therapy is unusual.^26^ It is unclear whether the development of a Balo‐like lesion in this case is associated with a new MS attack or the use of fingolimod. However, the possibility of an association between new BCS and fingolimod use needs further investigation.

## AUTHOR CONTRIBUTIONS

**Parisa Sharifi:** Conceptualization; formal analysis; writing – original draft; writing – review and editing. **Amir Moradi:** Conceptualization; formal analysis; writing – original draft. **Abdorreza Naser Moghadasi:** Conceptualization; formal analysis; writing – original draft; writing – review and editing.

## FUNDING INFORMATION

The authors received no funding for this study.

## CONFLICT OF INTEREST STATEMENT

The authors declare no conflict of interest regarding this article.

## ETHICS STATEMENT

The current study did not require ethical approval in accordance with local ethical guidelines. The study was conducted in accordance with Helsinki declaration and informed consent was obtained from patients to discuss or publish the details of their disease, their images, and their course of treatment.

## CONSENT

Written informed consent was obtained from patient to publish this report in accordance with the journal's patient consent policy.

## Data Availability

The data used to support the findings of this study are included within the article.

## References

[1] Rościszewska‐Żukowska I , Galiniak S , Bartosik‐Psujek H . Clinical characteristics of headache in multiple sclerosis patients: a cross‐sectional study. J Clin Med. 2023;12:3518. doi:10.3390/jcm12103518 37240624 PMC10219221

[2] Hauser SL , Cree BAC . Treatment of multiple sclerosis: a review. Am J Med. 2020;133:1380‐1390.e2. doi:10.1016/j.amjmed.2020.05.049 32682869 PMC7704606

[3] Chun J , Brinkmann V . A mechanistically novel, first oral therapy for multiple sclerosis: the development of fingolimod (FTY720, Gilenya). Discov Med. 2011;12:213‐228. 21955849 PMC3694567

[4] Frederick MC , Cameron MH . Tumefactive demyelinating lesions in multiple sclerosis and associated disorders. Curr Neurol Neurosci Rep. 2016;16:26. doi:10.1007/s11910-016-0626-9 26847090

[5] Ongphichetmetha T , Aungsumart S , Siritho S , et al. Tumefactive demyelinating lesions: a retrospective cohort study in Thailand. Sci Rep. 2024;14:1426. doi:10.1038/s41598-024-52048-w 38228919 PMC10791607

[6] Tzanetakos D , Vakrakou AG , Tzartos JS , et al. Heterogeneity of Baló's concentric sclerosis: a study of eight cases with different therapeutic concepts. BMC Neurol. 2020;20:400. doi:10.1186/s12883-020-01971-2 33138795 PMC7604966

[7] Baghbanian SM , Bakhshi A . Tumefactive Balo‐like concentric sclerosis in treatment with Fingolimod. Mult Scler Relat Disord. 2023;71:104321. doi:10.1016/j.msard.2022.104321

[8] Calabresi PA , Radue EW , Goodin D , et al. Safety and efficacy of fingolimod in patients with relapsing‐remitting multiple sclerosis (FREEDOMS II): a double‐blind, randomised, placebo‐controlled, phase 3 trial. Lancet Neurol. 2014;13:545‐556. doi:10.1016/s1474-4422(14)70049-3 24685276

[9] Kappos L , Cohen J , Collins W , et al. Fingolimod in relapsing multiple sclerosis: an integrated analysis of safety findings. Mult Scler Relat Disord. 2014;3:494‐504. doi:10.1016/j.msard.2014.03.002 25877062

[10] Ward MD , Jones DE , Goldman MD . Overview and safety of fingolimod hydrochloride use in patients with multiple sclerosis. Expert Opin Drug Saf. 2014;13:989‐998. doi:10.1517/14740338.2014.920820 24935480

[11] Algahtani H , Shirah B , Alassiri A . Tumefactive demyelinating lesions: a comprehensive review. Mult Scler Relat Disord. 2017;14:72‐79. doi:10.1016/j.msard.2017.04.003 28619436

[12] Nakayama M , Naganawa S , Ouyang M , et al. A review of clinical and imaging findings in Tumefactive demyelination. AJR Am J Roentgenol. 2021;217:186‐197. doi:10.2214/ajr.20.23226 34010036

[13] Pilz G , Harrer A , Wipfler P , et al. Tumefactive MS lesions under fingolimod: a case report and literature review. Neurology. 2013;81:1654‐1658. doi:10.1212/01.wnl.0000435293.34351.11 24097813

[14] Koudriavtseva T , Lorenzano S . A possible role of impaired cell‐mediated immunity in the pathogenesis of tumefactive demyelinating lesions. Mult Scler Relat Disord. 2017;18:184‐185. doi:10.1016/j.msard.2017.10.006 29141807

[15] Hardy TA , Reddel SW , Barnett MH , Palace J , Lucchinetti CF , Weinshenker BG . Atypical inflammatory demyelinating syndromes of the CNS. Lancet Neurol. 2016;15:967‐981. doi:10.1016/s1474-4422(16)30043-6 27478954

[16] Hardy TA , Miller DH . Baló's concentric sclerosis. Lancet Neurol. 2014;13:740‐746. doi:10.1016/s1474-4422(14)70052-3 24943346

[17] Moore GR , Neumann PE , Suzuki K , et al. Balo's concentric sclerosis: new observations on lesion development. Ann Neurol. 1985;17:604‐611. doi:10.1002/ana.410170614 4026231

[18] Karaarslan E , Altintas A , Senol U , et al. Baló's concentric sclerosis: clinical and radiologic features of five cases. AJNR Am J Neuroradiol. 2001;22:1362‐1367. 11498428 PMC7975204

[19] Okar L , Canibano B , Deleu D . Management of Baló Concentric Sclerosis with rituximab: a case study with long‐term follow‐up. Neuroimmunol Rep. 2023;4:100177. doi:10.1016/j.nerep.2023.100177

[20] Jolliffe EA , Guo Y , Hardy TA , et al. Clinical and radiologic features, pathology, and treatment of Baló concentric sclerosis. Neurology. 2021;97:e414‐e422. doi:10.1212/wnl.0000000000012230 34011576 PMC8362356

[21] Hall WA . Infectious lesions of the brain stem. Neurosurg Clin N Am. 1993;4:543‐551. 8353452

[22] Gavito‐Higuera J , Mullins CB , Ramos‐Duran L , Olivas Chacon CI , Hakim N , Palacios E . Fungal infections of the central nervous system: a pictorial review. J Clin Imaging Sci. 2016;6:24. doi:10.4103/2156-7514.184244 27403402 PMC4926551

[23] Arenas Vargas LE , Bedoya Morales AM , Rincón Carreño C , Espitia Segura OM , Penagos N . Balo's concentric sclerosis: an atypical demyelinating disease in pediatrics. Mult Scler Relat Disord. 2020;44:102198. doi:10.1016/j.msard.2020.102198 32531753

[24] Seewann A , Enzinger C , Filippi M , et al. MRI characteristics of atypical idiopathic inflammatory demyelinating lesions of the brain: a review of reported findings. J Neurol. 2008;255:1‐10. doi:10.1007/s00415-007-0754-x 18004634

[25] Sánchez P , Meca‐Lallana V , Vivancos J . Tumefactive multiple sclerosis lesions associated with fingolimod treatment: report of 5 cases. Mult Scler Relat Disord. 2018;25:95‐98. doi:10.1016/j.msard.2018.07.001 30056362

[26] Kania K , Ambrosius W , Kozubski W , Kalinowska A . Case report: Baló's concentric sclerosis‐like lesion in a patient with relapsing‐remitting multiple sclerosis treated with dimethyl fumarate. Front Neurol. 2022;13:891113. doi:10.3389/fneur.2022.891113 35677328 PMC9168072

